# Transposable Elements in Bats Show Differential Accumulation Patterns Determined by Class and Functionality

**DOI:** 10.3390/life12081190

**Published:** 2022-08-04

**Authors:** Nicole S. Paulat, Erin McGuire, Krishnamurthy Subramanian, Austin B. Osmanski, Diana D. Moreno-Santillán, David A. Ray, Jinchuan Xing

**Affiliations:** 1 Department of Biological Sciences, Texas Tech University, Lubbock, TX 79409, USA; 2 Department of Genetics, Rutgers, The State University of New Jersey, Piscataway, NJ 08854, USA; 3 Human Genetics Institute of New Jersey, Rutgers, The State University of New Jersey, Piscataway, NJ 08854, USA

**Keywords:** transposable elements, genome evolution, insertion preference

## Abstract

Bat genomes are characterized by a diverse transposable element (TE) repertoire. In particular, the genomes of members of the family Vespertilionidae contain both active retrotransposons and active DNA transposons. Each TE type is characterized by a distinct pattern of accumulation over the past ~40 million years. Each also exhibits its own target site preferences (sometimes shared with other TEs) that impact where they are likely to insert when mobilizing. Therefore, bats provide a great resource for understanding the diversity of TE insertion patterns. To gain insight into how these diverse TEs impact genome structure, we performed comparative spatial analyses between different TE classes and genomic features, including genic regions and CpG islands. Our results showed a depletion of all TEs in the coding sequence and revealed patterns of species- and element-specific attraction in the transcript. Trends of attraction in the distance tests also suggested significant TE activity in regions adjacent to genes. In particular, the enrichment of small, non-autonomous TE insertions in introns and near coding regions supports the hypothesis that the genomic distribution of TEs is the product of a balance of the TE insertion preference in open chromatin regions and the purifying selection against TEs within genes.

## 1. Introduction

Transposable elements (TEs), also known as mobile elements, are repetitive sequences of DNA that are able to mobilize in and across genomes through multiple mechanisms. TE transpositions not only introduce new TE sequences at the integration sites but can also introduce mutations because of DNA breaks/repairs during this process. Over time, TE transposition has the ability to change protein products, alter gene expression, and reconstruct genomic architecture [1,2,3,4,5,6,7,8,9].

Because of the dramatic impacts TEs can have, it is important to understand their insertion and accumulation patterns in genomes. Several hypotheses propose that TE transposition and integration can act as powerful drivers of genome evolution and facilitators of diversification [10,11,12]. Studies have also implicated TEs in regulatory innovations in developmental and immune pathways, as well as in human diseases [13,14,15,16,17,18,19]. Recent advances in sequencing technology and genomic databases allowed studies to gain detailed insights into the deep and widespread influence of TEs [20,21].

However, the spatial accumulation patterns of TEs in mammalian genomes have yet to be well established, although recent studies show TEs contribute to both 3D chromatin structure and the transcriptional regulation of nearby genes [7,8,9]. One hypothesis of TE accumulation states that TE activity is selected against in coding regions because of its potential destruction of genome functionality and integrity [22]. The converse school of thought is that TE activity is selected for in expressed regions because the loose chromatin structure allows for transposition mechanisms, and the “wound” nucleosome state inhibits transposition [23].

TE diversity across species can be substantial, and different classes of TEs also have distinct transposition mechanisms. These differences may impact how the insertion regions are affected [24,25]. Class I elements (retrotransposons) replicate using a copy-and-paste mechanism: the element is transcribed into an RNA intermediate (‘copy’), which is then reverse-transcribed and inserted (‘paste’) in another location in the genome [25]. Class II elements, in particular TIR-like DNA transposons, on the other hand, ‘cut’ themselves out of the genome with a TE-encoded transposase and insert (‘paste’) into a new location. Finally, rolling circle (RC) transposons such as Helitrons (also Class II) mobilize in a replicative fashion distinct from their fellow Class II elements and from retrotransposons [25]. The downstream impacts of these three classes could therefore be distinct. For example, retrotransposition of long interspersed elements (LINEs) and short interspersed elements (SINEs) requires two sequential single-strand nicks in the target site DNA for insertion, while the transposition of many DNA transposons requires double-strand cuts to at the target site [26,27]. Like LINEs, RC transposons utilize single nicks but across multiple DNA strands. Thus, the scale of impact is almost certain to vary across elements and across species, but the extent of these differences, and their related mechanisms, are not yet clear. This is especially true for mammals as the vast majority of TE research in this clade has been conducted in species that do not harbor active Class II elements.

Another area of interest is the non-random distribution of TEs across a genome. Several factors impact where TEs accumulate. Different TEs have different target site preferences, and even those with the same preference can accumulate in different genomic regions. For example, in mammals, retrotransposons such as LINEs and SINEs prefer AT-rich target sites, but LINEs accumulate in AT-rich regions while SINEs tend to accumulate in GC-rich regions [28,29]. There are many possible reasons for this pattern, including TE size, how effectively host defenses interfere with transposition, whether the target site is in open or closed chromatin, or selection against non-homologous recombination to remove existing insertions [23,28,30]. However, studies examining the factors affecting TE distribution patterns mainly focused on a few plant and animal models (*Arabidopsis*, maize, *Drosophila*, mouse, and human), so the universality of such patterns remains to be evaluated.

In this study, we examined the correlation between annotated TEs and functional regions of the genome to test the hypothesis that TE insertions are selected against in genic regions. We also analyzed the activity of TE insertions relative to their age. We leveraged the increased TE diversity observed in bats to determine if Class II elements exhibit distinct patterns relative to the more extensively studied Class I elements. The data show a depletion of all TEs in the coding sequence and reveal patterns of species- and element-specific attraction in the transcript region. Trends of attraction in the absolute and relative distance tests suggest significant TE activity in regions adjacent to genes. These data suggest that the accumulation of TEs is influenced by selective pressure against TEs in protein-coding sequences and against insertions in nucleosomal DNA. The type of TE also contributes to the selection pressure. The enrichment of small, non-autonomous TEs in introns and near coding regions supports the hypothesis that genomic locations of TEs are the product of a balance of the two competing selective pressures.

## 2. Materials and Methods

### 2.1. Species Selection and TE Annotation

Genomes of seven species of bats were included in this study (Table S1). Genome assemblies and gene annotation files of six bat species were downloaded from the Bat1K project (https://bds.mpi-cbg.de/hillerlab/Bat1KPilotProject/, accessed on 1 April 2022). The *Myotis lucifugus* genome assembly (GCA_000147115.1) and gene annotation were obtained from the UCSC genome browser (https://hgdownload.soe.ucsc.edu/goldenPath/myoLuc2/bigZips/myoLuc2.fa.gz, accessed on 1 April 2022; https://hgdownload.soe.ucsc.edu/goldenPath/myoLuc2/bigZips/genes/myoLuc2.ncbiRefSeq.gtf.gz, accessed on 1 April 2022). The CpG islands in each genome were identified based on the genome assembly using a hidden Markov model implemented in the program makeCGI (v 1.3.4) [31].

TE insertions were annotated with RepeatMasker v4.0.9 using a custom TE library [32]. This TE library is a combination of mammalian TE sequences that were available from RepBase as well as those manually curated by the Zoonomia project. The custom TE library is available as a supplemental file at https://doi.org/10.7282/00000212 (accessed on 1 August 2022), and all consensuses have been submitted to DFAM [33].

Raw RepeatMasker output was reformatted to BED format and split by TE class. TE classes were defined as LINE, SINE, LTR (long terminal repeat), DNA, and RC. TEs shorter than 100 base pairs (bps) were excluded from further analysis. A previous study showed that RC activities were exclusive to vesper bats among bat species in the past ~40 million years [34]. The small number of RCs annotated by RepeatMasker in species outside of the Vespertilionidae linkage were likely to be false positives and were therefore excluded from the analysis. Old and young TE subsets were determined based on approximate age. For the young group, TEs younger than ~25 million years (Mys) were selected; for the old group, TEs older than 40 Mys were selected. The age of the TEs was calculated based on species-specific mutation rates (Table S1) determined by consensus phylogeny branch lengths from Foley et al. [35] and median divergence estimates from TimeTree [36]. The TE selection was performed using filter_beds.py (https://github.com/davidaray/bioinfo_tools/blob/master/filter_beds.py, accessed on 1 April 2022).

### 2.2. TE Spatial Distribution Analysis

The GenometriCorr package’s suite (http://genometricorr.sourceforge.net/, accessed on 1 April 2022) was used to evaluate whether the spatial distribution of each TE group is independent of the positions of genes [37]. The three statistical tests were the Jaccard measure test, absolute distance test, and relative distance test. The significance of the tests was obtained through 100 permutations. The overall results of each test, based on all chromosomes with both TE and gene annotations, were used to determine if each TE group had a non-random spatial distribution in the bat genomes.

## 3. Results

### 3.1. TE Composition in Seven Bat Species

**Figure 1:**
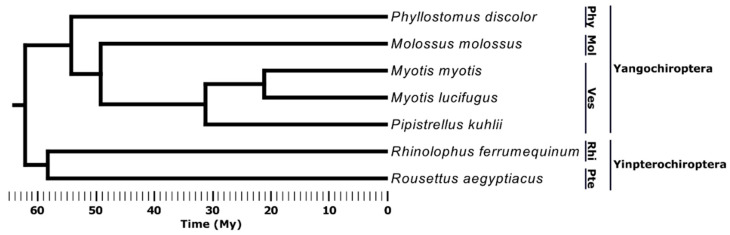
Phylogenetic tree of species in this study (based on [32]). Bat suborder is shown at far right, and family is indicated by three-letter abbreviations: Phy = Phyllostomidae, Mol = Molossidae, Ves = Vespertilionidae, Rhi = Rhinolophidae, Pte = Pteropodidae.

For this study, we selected seven bat species (Figure 1). Two features of bat lineages among mammals allowed us to compare the distribution of class I and class II TEs in a unique fashion. First, unlike most mammals and most other bats, DNA and RC transposons are active within the vesper bat lineage (family Vespertilionidae) [38,39,40,41]. Second, the pteropodid lineage of megabats (family Pteropodidae) has experienced a nearly complete cessation of activity from all types of TEs over the past 40 Mys [34,42,43].

**Figure 2:**
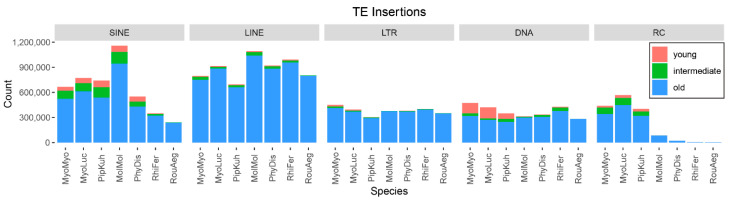
The number of annotated TEs for each species. Young: elements < 25 Mys old; Old: elements > 40 Mys old; Intermediate: elements between 25 and 40 Mys old. Species abbreviations: MyoMyo: *My. myotis*; MyoLuc: *My. lucifugus*; PipKuh: *Pi. kuhlii*; MolMol: *Mo. molossus*, PhyDis: *Ph. discolor*; RihFer: *Rh. ferrumequinum*; RouAeg: *Ro. aegyptiacus*.

First, we determined the TE composition in the seven bat genomes (Figure 2, Table S1). LINEs and SINEs are the most abundant TE classes in the seven species, a typical characteristic of mammals [34]. The numbers of SINEs and LINEs are the highest in the *Mo. molossus* genome (1,158,863 SINEs, 1,092,308 LINEs; Table S1). The composition of the genomes is relatively uniform for LINE, LTR, and DNA transposons but are variable for SINE and RC elements. For example, the genomes *My. myotis*, *My. lucifugus,* and *Pi. kuhlii* have about half a million RC insertions, while there are almost no RC insertions in other species.

Next, we examined the TE insertion dynamics in the seven genomes by counting the number of young (<25 Mys), intermediate (between 25 and 40 Mys), and old (>40 Mys) TEs in each genome (Figure 2). The proportion of young insertions is small, ranging from almost no activity in *Ro. aegyptiacus* to about 8% of all TEs in vesper bats. DNA transposons are the most active young elements in *My. myotis* and *My. lucifugus*, making up 55% and 52% of young insertions in their genomes, respectively. In *Ph. discolor*, *Mo. molossus*, and *Pi. kuhlii*, SINEs are the most active young elements and account for 80%, 80%, and 41% of all young insertions, respectively. The *Rh. ferrumequinum* genome contains a similar number of young SINE, LINE, and DNA elements (~10,000). As described previously, *Ro. aegyptiacus* have almost no recent TE activity.

**Figure 3:**
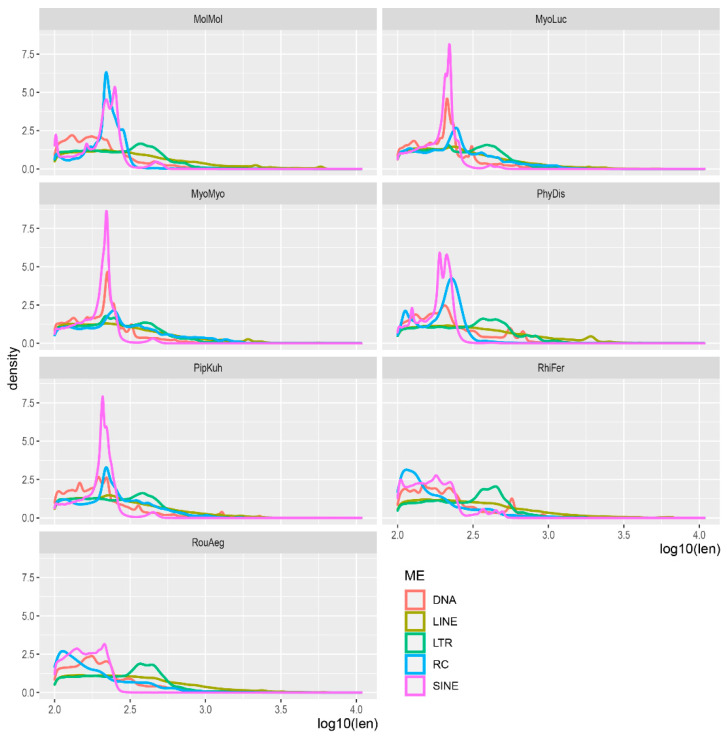
Size distribution of TEs in each species. The x-axis represents TE size (bps) on a log10 scale. Species abbreviations: MyoMyo: *My. myotis*; MyoLuc: *My. lucifugus*; PipKuh: *Pi. kuhlii*; MolMol: *Mo. molossus*, PhyDis: *Ph. discolor*; RihFer: *Rh. ferrumequinum*; RouAeg: *Ro. aegyptiacus*.

We then compared the size distribution of different TEs (Figure 3). Most SINEs, RCs, and DNA transposons are less than 300 bps in length. While the full length of LINE and LTR elements are 6–8 kbps in size, LINEs and LTRs in bat genomes showed a wide distribution. This is expected given that most LTR elements are solo-LTRs [44], while most LINEs are truncated [45].

### 3.2. TE Distribution Relative to Genic Regions and CpG Islands

We determined the relationship between genic regions, CpG islands (CGIs), and TE distributions across the genomes. For genic regions, we included the transcript, coding sequence (CDS), and the start codon position in our analysis. The transcript region is the broadest category tested, including introns, CDS, and untranslated regions. Compared to transcripts, the CDS and start codon represent the more conserved protein-coding regions. CGIs are regions of the genome with high levels of CpG dinucleotides and are generally associated with promoters [46]. We will collectively refer to the transcript, CDS, start codon, and CGI as genic features in the following text. For TEs, we tested three classes of retrotransposons (SINE, LINE, and LTR) and two classes of DNA transposons (DNA and RC). We focused on young (<25 Mys) and old (>40 Mys) TEs to determine the effect of insertion site preference and the effect of purifying selection against TEs on the TE genomic distribution, respectively. We applied Jaccard, absolute distance, and relative distance tests to examine the relationships between TEs and genic features. The Jaccard test determines the overlap between two sets of features (e.g., transcript and SINE) based on the ratio of the intersection to the union of the features. The absolute distance test measures the absolute minimum distance between two sets of features, while the relative distance test measures the average distance between the midpoints of two sets of features.

**Figure 4:**
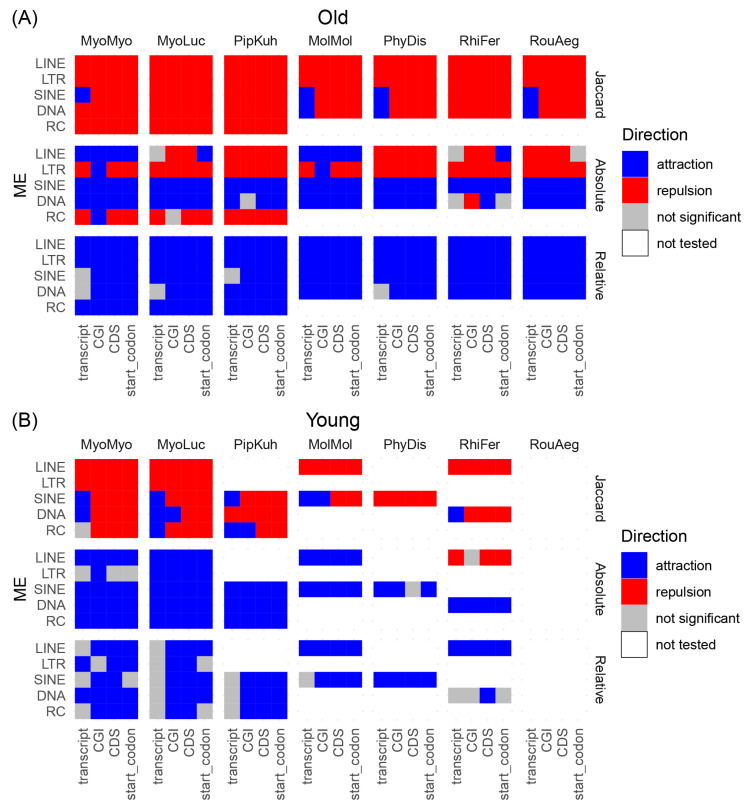
Relationship between TEs and genic features. Results from the Jaccard, absolute distance, and relative distance tests for old (**A**) and young (**B**) TEs. Minimum cutoff of 100 bp length for TE insertion, and 10,000 insertions per element per species. Attraction is indicated in blue, repulsion in red, no-significant in grey, and not-tested in white. Species abbreviations: MyoMyo: *My. myotis*; MyoLuc: *My. lucifugus*; PipKuh: *Pi. kuhlii*; MolMol: *Mo. molossus*, PhyDis: *Ph. discolor*; RihFer: *Rh. ferrumequinum*; RouAeg: *Ro. aegyptiacus*.

Figure 4 summarizes the correlations between TEs and genic features. Among old TEs (>40 Mys), the Jaccard test showed an overall pattern of repulsion among all TEs and gene features (Figure 4A). The only exceptions are SINE and DNA elements, which show enrichment of insertions (i.e., attraction) in the transcript of some species. The vast majority of SINE and DNA elements are non-autonomous and contain no open reading frames (ORFs). For the two types of autonomous retrotransposons, LTR and LINE, the Jaccard test showed depletion within all genic features in all species. Similarly, in vesper bat species that contain a high number of RC insertions, the Jaccard test showed depletion of RCs within all genic features. In contrast, the relative distance test showed an overall attraction between old TEs and gene features. The results of the absolute distance tests were intermediate between the Jaccard and relative distance test: while LINE, LTR, and RC elements showed depletion in genic features, SINE and DNA elements showed attraction patterns across the species, except for DNA elements and CGI in *Rh*. *ferrumequinum*. Taken together, these results suggest that, in general, old TEs are depleted within genic features but are in proximity of these features.

For young TEs (<25 Mys), because of the general low recent TE activities in bat species besides the vesper bats (Figure 2), we did not have enough insertions to test the interaction between some TEs and genic features. For groups that are large enough to be tested (i.e., >10,000 elements), the trends are generally consistent with old TEs in the Jaccard test: SINE and DNA elements are enriched within transcripts, while LINE and LTR elements are depleted in all genic features (Figure 4B). Interestingly, young RC elements in *My. lucifugus* and *Pi. kuhlii* showed attraction to the transcript, and DNA elements in *My. lucifugus* and SINEs in *Mo. molossus* showed attraction to CGI in the Jaccard test. For the absolute and relative distance tests, most of the tests showed enrichment of TEs within genic features, except for LINE elements in *Rh*. *ferrumequinum* (Figure 4B, Table S2).

## 4. Discussion

In this study, we examined the spatial relationships between all TE types and genic features in a variety of bats. Bats were chosen due to several clades that show unique TE characteristics when compared to other mammals. In particular, the recent activity of DNA transposons (Class II TEs) in vesper bats provides an opportunity to broaden the observations when compared to previous studies that had focused on mammals that have primarily experienced retrotransposon (Class I TEs) activities.

The near-unanimous attraction in the relative distance test demonstrates that TE insertions are closer to genic features than a random distribution. This result suggests a disproportionate accumulation of TEs in areas surrounding or within genes and it is consistent with the hypothesis proposed by Bennetzen [23], which states the openness of chromatin around genes, and especially around promoters, may play a role in promoting TE insertion in those regions.

In contrast, in the Jaccard test, where the intersection of features was evaluated, SINE and DNA transposons show differing results against transcripts compared to LINE and LTR elements. Both young and old LINE and LTR elements exhibit strong depletion in all genic features, suggesting selection against these insertions near genes, even soon after the insertion event. Conversely, both young and old SINEs and DNA transposons showed attraction against transcripts in several species. This is broadly consistent with trends shown in human and mouse genomes [28,30]. This different pattern may be explained by several differences among these TE groups. LINEs and LTRs are autonomous retrotransposons, and the full-length elements are large in size (6–8 kbps). As autonomous elements, LINEs and LTRs also harbor ORFs and numerous other functional features (e.g., promoters, polyadenylation sites, etc.). Therefore, the presence of a LINE or LTR insertion within or near a gene could have a dramatic impact on gene expression. On the other hand, SINE, DNA, and RC insertions are mostly small (~300 bps) and non-autonomous, with no ORFs and fewer potentially functional units than LINE/LTR insertions. The combination of smaller size and fewer potentially disruptive functional sequences may result in a weaker selective pressure against these types of insertions compared to LINE and LTR elements. To determine the impact of size on the distribution of LINE and LTR elements, we separated LINEs and LTRs into large (>500 bps) and small (≤500 bps) groups and tested their distributions within and outside of transcripts. The test showed that there are significantly fewer large LINEs/LTRs than small LINEs/LTRs within transcripts (Table S3, chi-square test with Yates’ continuity correction *p* < 10^−8^). These observations support the hypothesis of selection against large elements transposing into genic regions and the difficulty of successfully removing small elements very close to genes without impacting gene or regulatory region functions [22,28,47] (alternative explanation in [48]). Unlike human and mouse genomes, we also showed that DNA and young RC transposons have a similar distribution pattern to SINEs. These results may be due to the importance of chromatin accessibility for the insertion of these elements. Alternatively, shared distribution patterns of SINE and DNA transposons might suggest that functional impact, rather than the integration mechanism, plays a bigger role in determining the spatial distribution of TEs in the genome.

This body of data provides evidence to support the hypothesis that TE distribution is selected against in coding regions and enriched for upstream and downstream of genes. Further research is needed, given that our analyses are limited to bat species. In addition, this work did not extend to examining more complex relationships between genome structure, such as Z-DNA repeat sequences and TE integrations. Future studies can employ the same methods across the genomes of other bats and mammalian species to see if more nuanced patterns emerge between TE activity and genic regions. Continued research in the attraction and repulsion of TEs and genes relative to the age of the insertion may elucidate evolutionary relationships and TE influence on genome architecture.

## Data Availability

The genome annotation files for seven bat species used for the study, including gene annotation, CGI annotation, and TE annotation, are available at Rutgers University Community Repository (DOI: https://doi.org/10.7282/00000212, accessed on 1 August 2022).

## Supplementary Materials

The following supporting information can be downloaded at: https://www.mdpi.com/article/10.3390/life12081190/s1, Table S1: Genomic features; Table S2: GenometriCorr results; Table S3: Distribution of large and small LINEs/LTRs in transcripts.
